# MicroRNA410 Inhibits Pulmonary Vascular Remodeling via Regulation of Nicotinamide Phosphoribosyltransferase

**DOI:** 10.1038/s41598-019-46352-z

**Published:** 2019-07-09

**Authors:** Hui Gao, Jiwang Chen, Tianji Chen, Yifang Wang, Yang Song, Yangbasai Dong, Shuangping Zhao, Roberto F. Machado

**Affiliations:** 1 Department of Medicine, University of Illinois at Chicago, Chicago, IL 60612 USA; 2 Department of Obstetrics and Gynecology, Union Hospital, Tongji Medical College, Huazhong University of Science and Technology, Wuhan, 430022 China; 3 Department of Pediatrics, University of Illinois at Chicago, Chicago, IL 60612 USA; 4 Institute for Genome Sciences, University of Maryland School of Medicine, Baltimore, MD 21201 USA; 5 Division of Pulmonary, Critical Care, Sleep, and Occupational Medicine, Department of Medicine, Indiana University, Indianapolis, IN 46202 USA

**Keywords:** Respiratory tract diseases, Translational research

## Abstract

Nicotinamide phosphoribosyltransferase (NAMPT) upregulation in human pulmonary artery endothelial cells (hPAECs) is associated with pulmonary arterial hypertension (PAH) progression and pulmonary vascular remodeling. The underlying mechanisms regulating NAMPT expression are still not clear. In this study, we aimed to study the regulation of NAMPT expression by microRNA410 (miR410) in hPAECs and explore the role of miR410 in the pathogenesis of experimental pulmonary hypertension. We show that miR410 targets the 3′ UTR of NAMPT and that, concomitant with NAMPT upregulation, miR410 is downregulated in lungs of mice exposed to hypoxia-induced pulmonary hypertension (HPH). Our results also demonstrate that miR410 directly inhibits NAMPT expression. Overexpression of miR410 in hPAECs inhibits basal and VEGF-induced proliferation, migration and promotes apoptosis of hPAECs, while miR410 inhibition via antagomirs has the opposite effect. Finally, administration of miR410 mimics *in vivo* attenuated induction of NAMPT in PAECs and prevented the development of HPH in mice. Our results highlight the role of miR410 in the regulation of NAMPT expression in hPAECs and show that miR410 plays a potential role in PAH pathobiology by targeting a modulator of pulmonary vascular remodeling.

## Introduction

Pulmonary arterial hypertension (PAH) is a lethal vasculopathy of complex etiology that involves remodeling of distal pulmonary arteries leading to elevation of pulmonary vascular resistance. This process results in right ventricular (RV) failure and death^1^. There is still no cure for this disease. Thus, there is an imperative need to explore novel interventional pathways for PAH treatment.

Nicotinamide phosphoribosyl transferase (NAMPT), also named visfatin and pre-B cell colony-enhancing factor (PBEF), is a pleiotropic protein possessing extracellular proinflammatory cytokine-like activity and intracellular enzymatic activity as a phosphoribosyltransferase^2–8^. Our previous work demonstrated that NAMPT mediates pulmonary vascular remodeling and that inhibition of NAMPT activity is a potential therapeutic target for pulmonary arterial hypertension^9^. Specifically, we showed that NAMPT levels in human pulmonary artery endothelial cells (hPAECs) were upregulated in patients with PAH and that NAMPT promoted proliferation of hPAECs and human pulmonary artery smooth muscle cells (hPASMCs) via autocrine and paracrine effects, respectively^9^. The mechanisms for increased PAEC NAMPT expression in PAH are not known.

As a new class of posttranscriptional regulators of many cardiac and vascular diseases^10^, microRNAs (miRs) are a group of endogenous, small (approximately 22 nucleotides) non-coding RNAs which regulate gene expression^11^. Several studies have identified miRs associated with PAH and with experimental models of pulmonary hypertension^12–14^. They may be involved in the control of almost all physiological and pathological cellular processes, including several pathways relevant to pulmonary vascular remodeling. We hypothesized that miRNAs regulate NAMPT expression and could, in turn, affect proliferation, migration and apoptosis of PAECs. These specific miRs may also be therapeutic targets for PAH.

## Results

### MiR410 targets the UTR of NAMPT in hPAECs

**Figure 1:**
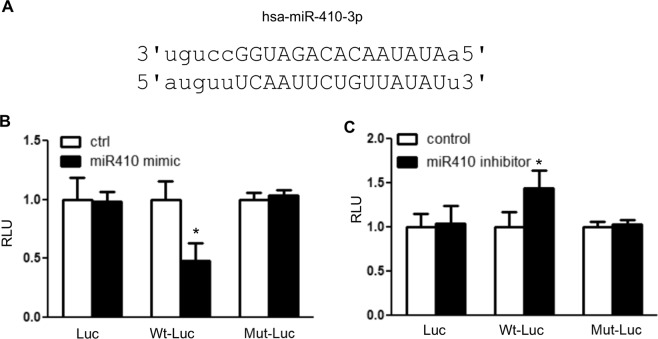
miR410 targets the 3′UTR of NAMPT in hPAECs. (**A**) The putative binding site of miR-410 at the 3′-UTR of NAMPT; (**B**) hPAECs were co-transfected with the pGL3 promoter, Wt-Luc or Mut-Luc reporter and miR-410 mimics or corresponding controls. (**C**) miR-410 inhibitors or corresponding controls were co-transfected with the pGL3 promoter, Wt-Luc or Mut-Luc reporter in hPAEC, and the luciferase activity was presented. N = 6 for each graph. *P < 0.05, relative to their corresponding controls.

We performed *in silico* analysis of the human NAMPT 3′UTR to predicted miR response elements. We found 5 potential miRs, including miR-410-3p as the highest predicted target of NAMPT with a high probability of down-regulation (mirSVR score: −1.2151; phastCons score: 0.7995). Figure 1A represents the hsa-miR410-3p putative binding site of miR410 within the 3′-UTR of NAMPT. To determine the interaction of miR410 with the NAMPT 3′-UTR, we overexpressed or inhibited miR410 expression using human miR410 mimics or inhibitors, respectively, in hPAECs co-transfected with a sequence-verified clone of NAMPT-3′UTR reporter vector designed for microRNA target validation. Overexpression of miR410 reduced luciferase activity due to NAMPT-3′UTR binding (Fig. 1B), while transfection of miR410 inhibitors increased the luciferase activity of Wt-luc (Fig. 1C). These effects were not demonstrated in the blank PGL-3 vector and a construct mutated in the predicted miR410-3p binding site.

### VEGF downregulates miR410 expression and upregulates NAMPT levels in hPAECs

**Figure 2:**
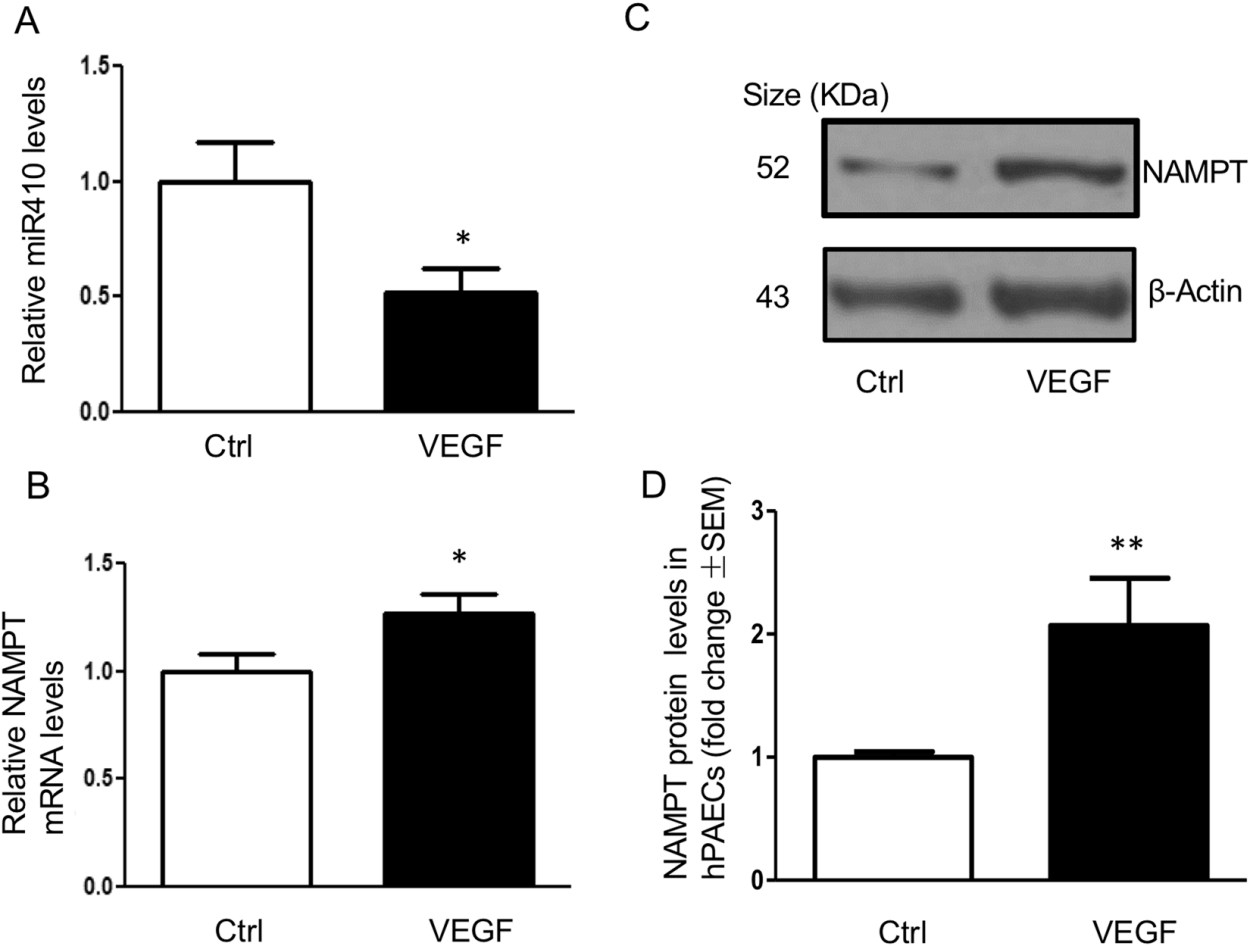
VEGF promotes NAMPT expression and down-regulates miR410 levels in hPAECs. (**A**) VEGF (50 ng/ml) downregulates miR410 levels in hPAECs after 24-hr exposure; (**B**) VEGF increases NAMPT mRNA levels in hPAECs after 24-hr exposure; (**C**,**D**) VEGF increases NAMPT protein levels in hPAECs after 24-hr exposure. Experiments were completed in triplicate. *P < 0.05, **P < 0.01, relative to controls.

Vascular endothelial growth factor promotes PAEC proliferation and is a major contributor to PAH pathobiology^15^. Incubation of hPAECs with VEGF (50 ng/ml) for 24 hours significantly decreased the expression of miR410 (Fig. 2A), and increased NAMPT mRNA and protein levels (Fig. 2B–D).

### MiR410 is down-regulated in hypoxia-induced pulmonary hypertension (HPH)

**Figure 3:**
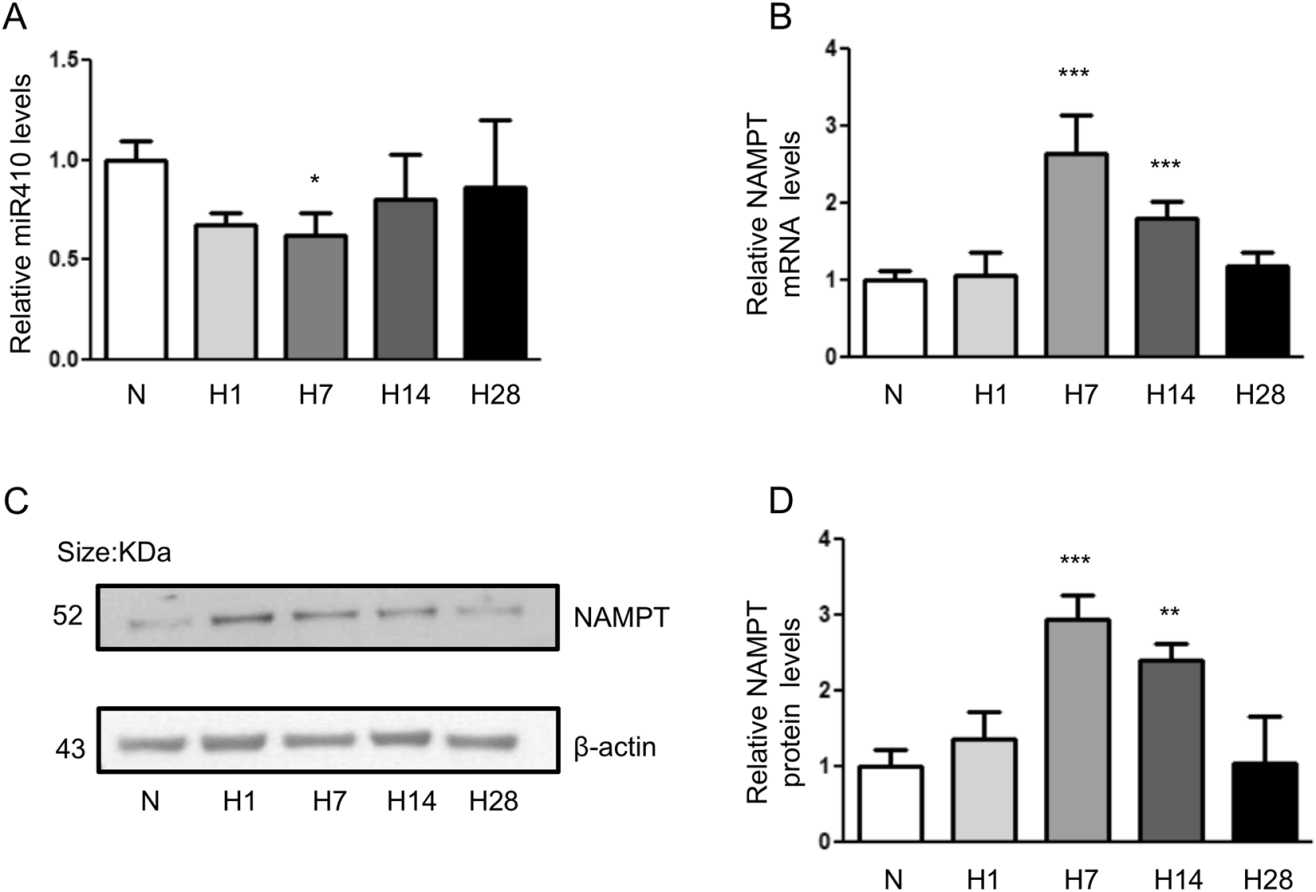
miR-410 is down-regulated in mouse lungs during the progression of experimental hypoxia-mediated pulmonary hypertension. (**A**) Time course of miR410 levels in mouse lungs. (**B**) NAMPT mRNA levels in mouse lungs significantly increased at Day 7 and Day 14 after hypoxia; (**C**,**D**) time course of NAMPT protein levels in mouse lungs. The significance was evaluated by comparing to normoxia. *P < 0.05; **P < 0.01; ***P < 0.001.

We exposed mice to HPH to investigate the expression of miR410(Fig. 3A) as well as NAMPT mRNA (Fig. 3B) and protein levels (Fig. 3C,D) following 1, 7, 14, and 28 days of 10% O_2_ exposure. The whole lung expression of miR410 significantly decreased following hypoxia exposure in day 7 (ANOVA: p = 0.05, Tukey post-hoc: p < 0.05), increasing close to baseline levels after days 14 and 28. In contrast an inverse pattern was demonstrated for NAMPT mRNA and protein levels during hypoxia exposure (p = 0.0002, Tukey post-hoc: p < 0.05).

### MiR410 regulates the expression of NAMPT in hPAECs

**Figure 4:**
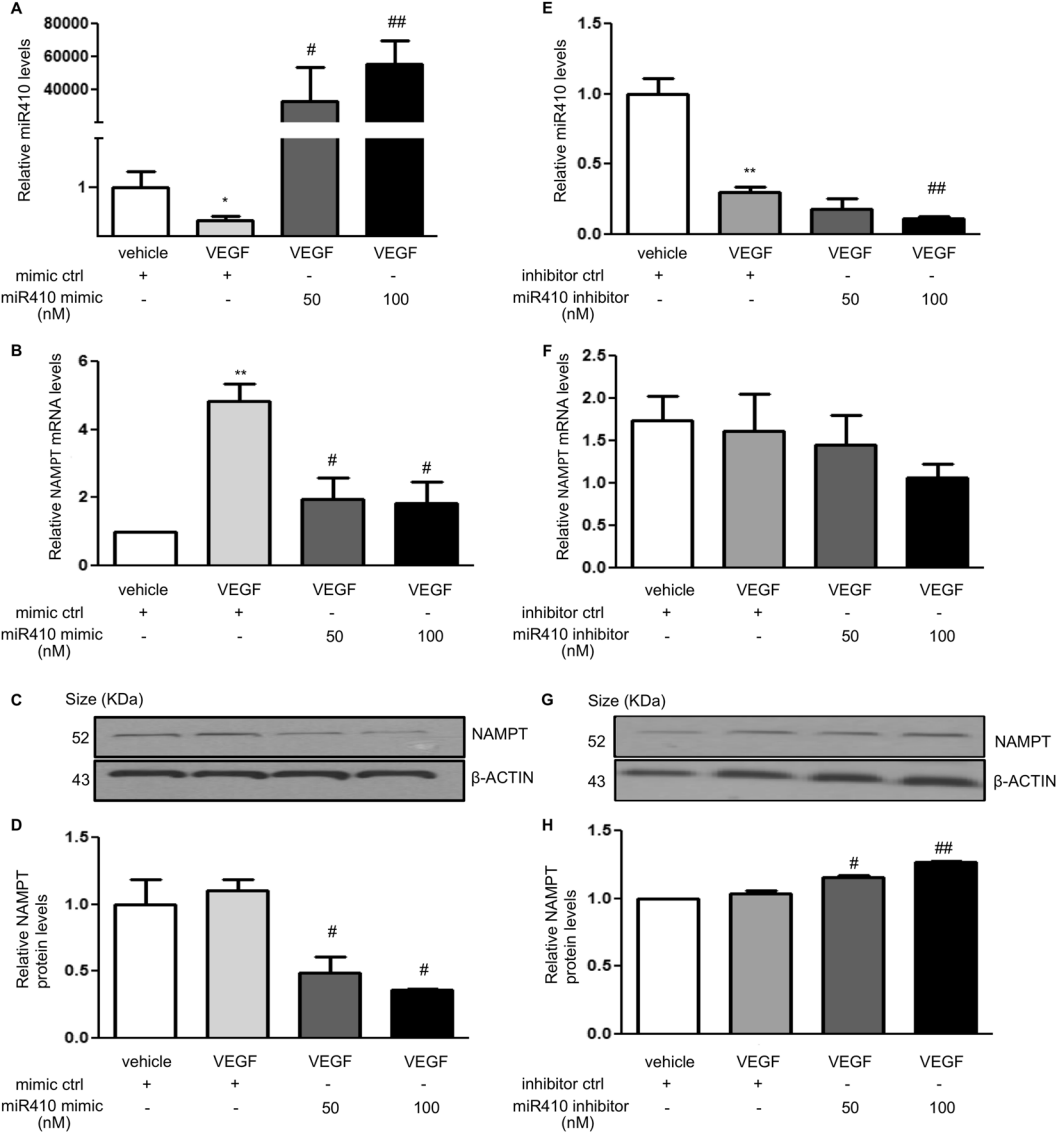
miR410 overexpression inhibits VEGF-induced NAMPT expression in hPAECs. VEGF decreases miR410 levels and increases NAMPT levels in hPAECs after 48-hr incubation. Overexpressing miR410 significantly increases miR410 levels in hPAECs (**A**), reduces NAMPT mRNA (**B**) and protein levels (**C**,**D**). Blocking miR410 via a specific inhibitor decreases miR410 levels (**E**), does not significantly change NAMPT mRNA levels and increases NAMPT protein levels (**G**,**H**). *P < 0.05; **P < 0.01 relative to vehicle; ^#^P < 0.05; ^##^P < 0.01, relative to 50 nM group.

To test if miR410 may be a negative regulator of NAMPT expression, we transfected hPAECs with miR410 mimics (Fig. 4A) and inhibitors (Fig. 4E), respectively. After transfection with miR410 mimics, NAMPT protein and mRNA levels significantly decreased (Fig. 4B–D). While after transfection with miR410 inhibitors, NAMPT mRNA (Fig. 4F) levels did not statistically increase, NAMPT protein levels significantly increased (Fig. 4G,H).

### MiR410 regulates the proliferation and migration of hPAECs

**Figure 5:**
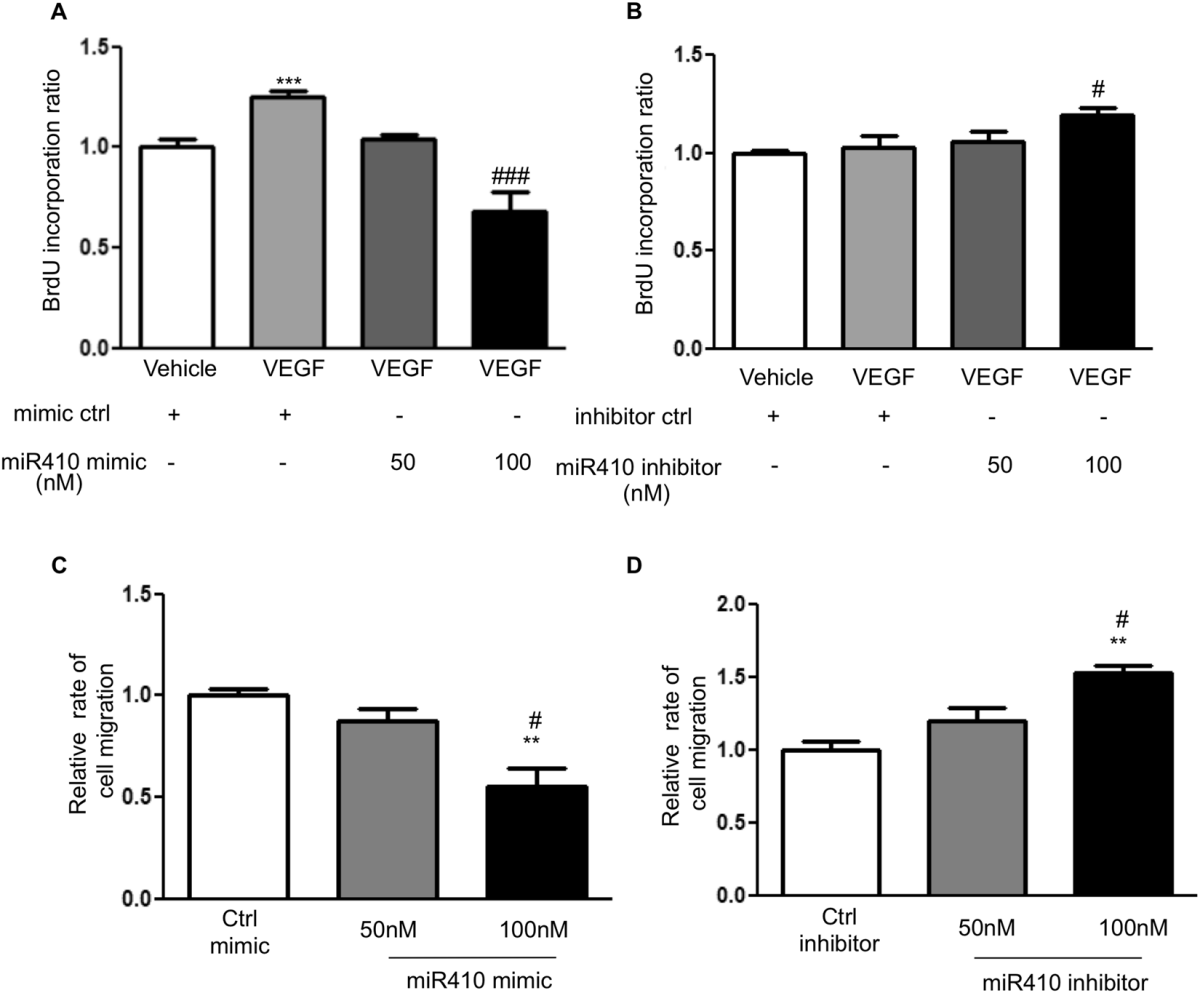
miR410 regulates VEGF-induced hPAECs proliferation and migration. Overexpression of miR410 in hPAECs (100 nM) significantly (**A**) decreases VEGF-mediated hPAEC proliferation in comparison to control while overexpression of miR410 inhibitors (100 nM) (**B**) increases VEGF-mediated hPAEC proliferation. Overexpression of miR410 in hPAECs (100 nM) significantly decreases (**C**) migration while overexpression of miR410 inhibitors (100 nM) significantly increases (**D**) VEGF-mediated hPAEC migration. **P < 0.01; ***P < 0.001, relative to vehicles; ^#^P < 0.05, ^###^P < 0.001, relative to 50 nM group.

To investigate whether modulation of miR410 levels could alter the proliferation and migration of hPAECs, we transfected hPAECs with miR410 mimics or inhibitors in the absence or presence of VEGF stimulation. Results indicated statistically significant differences between the average levels in the four groups (ANOVA, p = 0.026). Overexpression of miR410 mimics reduced the proliferative capacity, and the reduction was significant with 100 nm miR410 treatment (post post-hoc test, p = 0.02) (Fig. 5A), while overexpression of miR410 inhibitors significantly increased hPAECs proliferation at 100 nm (post-hoc test, p = 0.03) (Fig. 5B). Under the same conditions, a similar trend was found for hPAEC migration (Fig. 5C,D).

### MiR410 promotes hPAECs apoptosis

**Figure 6:**
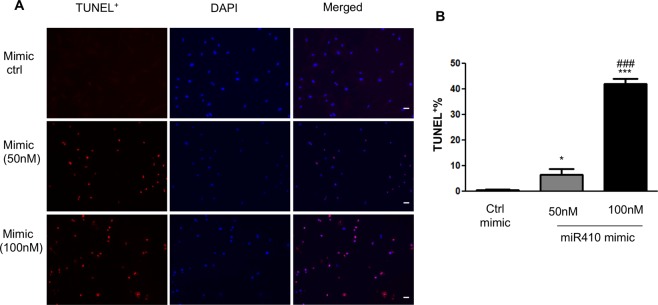
miR410 overexpression increases hPAECs apoptosis. (**A**) Representative images of TUNEL positive cells (red), nucleus was stained by DAPI, and percentage of TUNEL positive cells was quantified in panel B. *P < 0.05, ***P < 0.001 relative to control; ^###^P < 0.001 relative to 50 nM group. Size bar: 20 µm.

Since we have previously shown that NAMPT promotes resistance to apoptosis^9^, we investigated whether miR410 promotes hPAEC apoptosis. As shown in Fig. 6A,B, miR410 over expression induced hPAEC apoptosis in a dose dependent manner.

### *In vivo* MiR410 overexpression attenuates the progression of HPH

**Figure 7:**
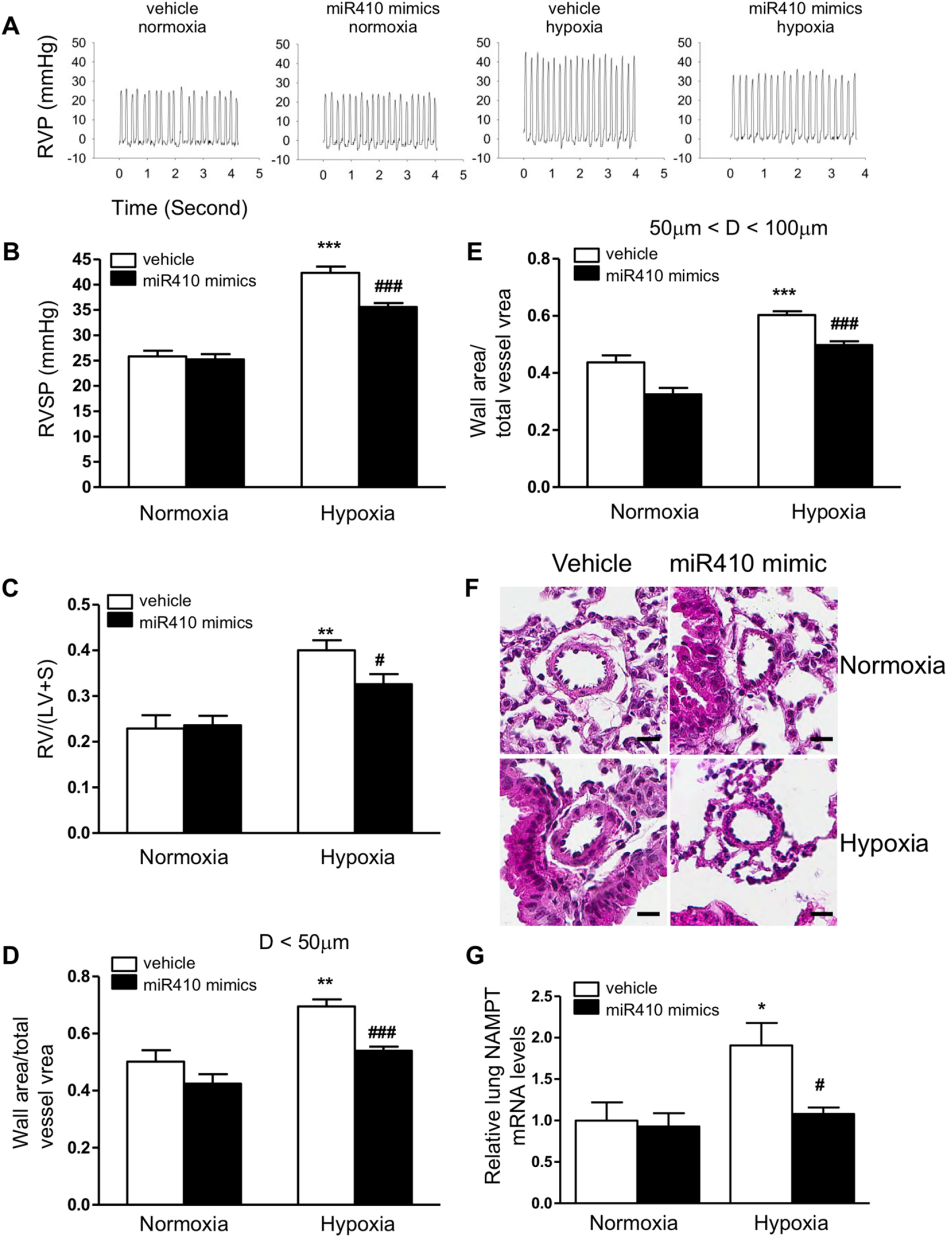
miR410 overexpression *in vivo* attenuates the development of HPH in mice. Relative to controls, mice in the HPH model receiving systemic miR410 mimics (6.8 mg/kg b.w.) develop less severe (**A**,**B**) RVSP elevation, (**C**) RVH, and (**D**,**E**) pulmonary vascular remodeling in arteries with diameters <50 µm and 50–100 µm, respectively. (**F**) Representative histological images of small pulmonary arteries in normoxia and hypoxia, with and without treatment with systemic miR-410 mimics. (**G**) Systemic miR410 mimic administration significantly decreases NAMPT mRNA levels in whole lung tissues from mice with HPH relative to control (p = 0.035, Wilcoxon rank sum test). Scale bar = 20 µm. *P < 0.05; **P < 0.01.

**Figure 8:**
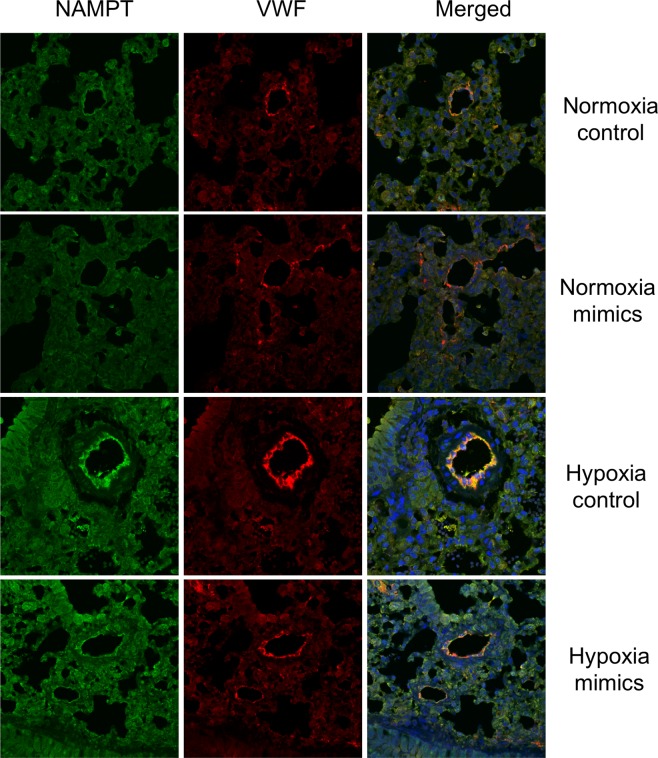
miR410 overexpression *in vivo* decreases PAEC NAMPT levels. Immunofluorescence staining demonstrates that hypoxia-induced NAMPT protein expression in pulmonary artery endothelial cells is reduced in miR410 mimic-treated mice relative to controls. Size bar = 20 µm.

We delivered control non-targeted or miR410 mimics via retro-orbital injection to mice weekly on days 7, 14, 28 and 35 in animals exposed to normoxia or 10% hypoxia. As seen is Supplemental Fig. 1, delivery of micro RNA mimic significantly increased the lung expression of miR410 in both normoxia and hypoxia. In the hypoxia group, mice receiving miR410 mimics were found to have significant attenuation of RVSP elevation and the degree of RVH when compared to controls (Fig. 7A–C). Hypoxia-induced pulmonary vascular remodeling was also attenuated by miR410 mimic treatment (Fig. 7D–F). No significant changes in these parameters occurred in the normoxic group of animals. In the hypoxia group, NAMPT mRNA levels in lung tissues were significantly decreased by administration of miR410 mimic when compared to control. This effect was not seen in the normoxia group (Fig. 7G, Wilcoxon rank sum test, p = 0.35). NAMPT levels in PAECs were significantly decreased in mice receiving miR410 mimics in both normoxic and hypoxic conditions (Fig. 8).

## Discussion

In this study, we investigated the contribution of miR410 to pulmonary vascular remolding *in vitro* and *in vivo*. This work expands and complements our previous study^9^, where we found that NAMPT was upregulated in the plasma, lungs, and PAECs isolated from patients with PAH and in PAECs from different rodent models of PH. Further, we have shown that PAECs from patients with PAH were more proliferative and secrete more NAMPT, which promotes PASMC proliferation in a paracrine fashion^9^. In this study we identified miR410 as a potential regulator of NAMPT expression. We found that miR-410 is downregulated by VEGF *in vitro* and by hypoxia *in vivo* and that these changes are coincident with increased NAMPT expression. In addition, we show that miR-410 inhibits hPAEC proliferation and migration while promoting hPAEC apoptosis and that miR410 overexpression attenuates the development of experimental HPH while reducing the lung overexpression of NAMPT in this model. Thus, our results describe a novel role for miR410 in controlling the proliferation, apoptosis and migration of hPAECs, and affecting pulmonary vascular remodeling via regulation of NAMPT expression.

In PAECs from patients with PAH, cancer-like phenotypes such as uncontrolled vascular cell proliferation, apoptosis resistance, and a shift from oxidative phosphorylation towards glycolysis as an energy source have been well documented^16,17^. In this context, miR410 may target other genes that may play active roles in the development of PAH. Several studies suggest that miR410 may have cell specific roles in promoting proliferative and pro survival pathways. Mussnich *et al*. demonstrated that miR410 is downregulated in gonadotroph tumors and targets Cyclin B1 (CCNB1) at protein and mRNA levels, decreasing cell proliferation^18^. Similar findings demonstrating a tumor suppressor role for miR410 were also shown in lung cancer, osteosarcoma, colorectal cancer, breast cancer, cholangiocarcinoma, glioma, oral squamous cell carcinoma and pancreatic cancer^19–25^. On the other hand, Li *et al*. have showed that miR410 targets bromodomain-containing protein 7 (BRD7) and promotes progression of non-small cell lung cancer (NSCLC) via modulation of cell proliferation, migration, and invasion due to downregulation of BRD7 leading to an increase of Akt phosphorylation^26^. Studies have also described oncogenic properties of miR410 in the context of other malignancies^27–29^. Finally, miR410 has been shown to downregulate IL-10 expression in T cells via suppression of the transcriptional activity of STAT^30^, a transcriptional factor known to be involved in PAH pathobiology^31^.

The exact mechanistic role of VEGF and VEGF-dependent signaling in PAH is not completely understood but abnormalities in this pathway have been are well documented in human and experimental pulmonary hypertension^32,33^. Our data suggest that VEGF downregulates miR410 expression which in turn influences NAMPT expression and PAEC proliferation, migration and apoptosis. Interestingly, miR410 suppresses VEGF expression through interaction with the 3′UTR of the VEGF messenger RNA and overexpression of a miR-410 mimic effectively suppresses VEGF expression *in vitro* and prevents retinal angiogenesis in a model of retinal neovascularization^34^. Similar findings were also reported in osteosarcoma cell lines where the expression of VEGF and miR-410 where inversely correlated and miR-410 overexpression resulted in decreased osteosarcoma cell proliferation and migration and increased apoptosis^20^. Taken together, these data suggest the presence of a feedback loop regulating the expression of these two mediators. Further studies are needed to test whether these interactions involve NAMPT directly or indirectly.

MiR-410 may also potentially affect right ventricular remodeling and dysfunction associated with PAH. Clark and Naya have shown that overexpression of miR-410 promotes cardiomyocyte proliferation by targeting Cited2; a transcriptional coactivator that has been linked to important developmental processes in the heart^35^. In a follow up study, the same group demonstrated that together with miR-495, miR-410 expression was increased in animal models of myocardial infarction, left ventricular hypertrophy and dystrophic cardiomyopathies; and that silencing of miR-410 and/or miR-495 blunted cardiomyocyte hypertrophy *in vitro*^36^.

Micro-RNAs are candidate therapeutic targets and biomarkers for PAH (as reviewed by Pullamsetti *et al*.^37^ and Huston *et al*.^38^). For instance, in one of our previous studies^14^, miR1 has been identified as an important regulator of SphK1 expression in PASMCs and a potential therapeutic target for the treatment of PH. Our *in silico* analyses, however, do not suggest that miR1 or other recently reported PAH-related micro RNAs are predicted to regulate NAMPT expression. As previously discussed, miR-410 regulates VEGF expression and similar interactions with other genes and micro RNAs involved in PAH are also possible.

Our results suggest that miR-410 has a role in regulating pulmonary vascular remodeling. There are, however, several limitations in the current study. First, validation of miR410 is lacking in samples from patients with PAH. Second, in this study only a mouse model of hypoxia mediated PH model was used. This model induces a mild PH phenotype, and we have not tested the effect of overexpressing miR410 in rodent models of severe PH. Evaluation of miR410 expression levels in PAH patients’ blood, lung tissues and PAECs and the effect of overexpressing miR410 in rodent models of severe PH warrant further investigations.

In summary, we show that miR410 plays a potential role in PH pathobiology by targeting NAMPT, a modulator of pulmonary vascular remodeling. Overexpression of miR410 in the pulmonary circulation may have a therapeutic effect in PAH.

## Materials and Methods

### Bioinformatics analysis and miRNA prediction

*In silico* analysis for microRNA target prediction was completed via the MicroRNA.org target prediction resource, utilizing miRSVR < −1.10 and PhastCons scores >0.70. and with further 3′- UTR sequence target sites prediction using miRDB.org as we have previously described^14^.

### Human cell lines

A primary Human pulmonary artery endothelial cell (hPAEC) line from Lonza (CC-2530) was used in this study. Vascular endothelial growth factor (VEGF, 293-VE, R&D System) was used at a final concentration of 50 ng/ml. MiR410 mimics, inhibitors and controls (50–100 nM, Qiagen) were transfected to hPAECs using lipofectamine (Thermo Fisher Scientific) per manufacturer’s instructions.

### Transient transfections and reporter assays

Mir Vana^TM^ miRNA-410-3P mimics or inhibitors and their corresponding negative controls were used for all microRNA transfection studies (Thermo Fisher Scientific). To construct the luciferase- NAMPT 3′-UTR (Wt-luc) reporter plasmid, a 227 bp 3′-UTR of human NAMPT gene containing the predicted miR410-3p binding site was amplified from human genomic DNA and inserted into downstream of the luciferase reporter gene in the pGL3-Promoter vector (Promega) through the XbaI endonuclease restriction site. We mutated the predicted miR410-3p binding site on the Wt-luc reporter plasmid to generate the Mut-luc reporter using the QuikChange Lightning Site-Directed Mutagenesis Kit (Stratagene, La Jolla, CA). All constructs were sequenced for confirmation.

hPAECs were plated in 12-well plates, and co-transfected with 1 μg of either Wt-luc or Mut-luc reporter plasmid, 50 ng of Renilla reporter plasmid and 50 pmole control or miR410-3p mimics/inhibitors using Lipofectamine 2000 reagent (Invitrogen). The cells were lysed twenty-four hours after transfection, and the luciferase activity was measured using Dual-Luciferase Reporter Assay System (Promega) on GloMax®-96 Microplate luminometer (Promega). Relative luciferase activities were calculated by comparing the firefly/renilla luciferase ratio.

### Western blot analysis

As we have previously described^9,14,39^, lung tissues were perfused with PBS to remove blood and homogenized using RIPA buffer (Sigma) supplemented with protease and phosphatase inhibitor cocktails (Calbiochem). Cells were washed with PBS and protein was extracted using the same modified RIPA buffer solution. Proteins (10–25 μg) were separated on Mini-Protean TGX precast gels (Bio-Rad), transferred to nitrocellulose membranes, and blocked in 5% nonfat milk. Membranes were then probed with primary and secondary antibodies and bands visualized by ECL (Pierce) per manufacturer’s instructions. Densitometry was used to quantify protein levels using ImageJ software, and expression levels were normalized to β-actin. The primary antibodies used were NAMPT, and HRP-conjugated β-actin (Cell Signaling Technology). An anti-rabbit IgG HRP-linked secondary antibody was used (Cell Signaling Technology).

### Cell proliferation

Cell proliferation was determined using a 5-bromo-2’-deoxyuridine (BrdU) incorporation assay (QIA58; Calbiochem) per manufacturer’s instructions, as we have previously described,^9,14,39^. Starting cell densities of hPAEC 5,000 cells/well were used in a 96-well format.

### Cell migration assays

Cell migration was determined using a quantitative transwell assay as previously described^9^. In brief, 1 × 10^5^ cells were added into a transwell insert with 8-μm pores in 1 ml of basal medium. Cells were then incubated with miR410 mimic/mimic control or inhibitor/inhibitor control for 24 hr prior to transferring to the transwell insert. Cells were fixed and stained using 0.5% crystal violet staining solution, and imaged. Five random fields were imaged per sample to obtain a total cell count. Migrated cells were scraped away from the top of the filter and the migrated bottom layer of cells were imaged and quantified. Relative rate of cell migration was calculated as the migrated cell numbers on the filter normalized by the control group.

### *In Situ* BrdU- Red TUNEL assay

We performed TdT-UTP nick end labeling (TUNEL) method to label 3′-end of fragmented DNA of the apoptotic PASMCs. Three groups of cells cultured in 6-well plates were treated as described in the mitochondrial depolarization assay, then fixed with 4% paraform phosphate buffer saline, rinsed with PBS, then used *in situ* BrdU-Red DNA fragmentation (TUNEL) cell apoptosis detection kit (Abcam®, Cambridge, MA). The percentage of positive cells to whole PAECs, identified as apoptosis indices (AI), was calculated.

### MiRNA PCR analysis and quantitative real-time reverse transcription PCR (qRT-PCR)

Total RNA containing miRNA was isolated from hPASMC and lung tissue samples using QIAzol lysis reagent and column purified using a miRNeasy kit (Qiagen). After quantification with Nanodrop 2000 spectrophotometer (ThermoScientific, Rockford, IL), miRNAs were reversely transcribed using a RT2 miRNA First Strand Kit (SABiosciences, Frederick, MD). For qRT-PCR analysis of miRNA expression, a poly (A) tail was first added to the 3′-end of miRNAs using a Poly (A) Polymerase Tailing Kit (Epicentre Biotechnologies, Madison, WI). Poly (A) tailed-miRNAs were then reversely transcribed using M-MLV Reverse Transcriptase (Invitrogen, Grand Island, NY) with a poly (T) adaptor, which includes a poly (T) sequence and a sequence complementary to the universal primer used in following qRT-PCR analysis. SNORD44, SNORD47 and SNORD48 were used as internal controls. The expression of mRNAs was determined using specific TaqMan primer assays (Applied Biosystems) with GAPDH used as an internal control. Real-time PCR analysis was performed using a CFX384 system (Bio-Rad), and relative changes in mRNA and miRNA expression were calculated after normalization to their respective internal controls using the comparative Ct method.

### Animal model of hypoxia induced pulmonary hypertension

All animal procedures were reviewed and approved by the University of Illinois at Chicago Animal Care and Use Committee. All experiments were performed in accordance with relevant guidelines and regulations.

In the mouse model of hypoxia-mediated pulmonary hypertension (HPH), 8-wk old male C57BL/6 mice were exposed to normoxia or hypoxia (10% O_2_) for 1d-4wks (n = 3–5/group). HPH development was assessed by measuring right ventricular systolic pressure (RVSP) via a pressure transducer catheter (Millar) as a surrogate of pulmonary artery pressure, right ventricular hypertrophy as a weight ratio of the right ventricle divided by the sum of left ventricle and septum (RV/LV + S)^9,14,39^. Paraffinized lung tissue sections were stained with hematoxylin and eosin. Vessel thickness was measured using Aperio ImageScope software, approximately 20 muscular arteries with diameters 50–100 μm or less than 50 μm per lung section. Pulmonary vascular remodeling was calculated as ([external vessel area – internal vessel area]/external vessel area), as previously described^39^.

In reversal studies, mice were exposed to normoxia or hypoxia for five weeks (n = 6/group). Animal-grade mirVana miRNA mimics or negative miRNA control (Thermo Fisher Scientific) were prepared with Invivofectamine 3.0 reagent (Thermo Fisher Scientific) per manufacturer’s instructions and injected to mice retro-orbitally (6.8 mg/kg body weight) after one-week normoxia or hypoxia (10% O2) exposure, and then continued for another four weeks. Injections were repeated once per week during the experimental period. One day after the final injection, we assessed HPH development by measuring RVSP, RVH, and pulmonary arterial remodeling as described above. Lung, heart, and blood were isolated for further analysis.

### Lung tissue immunofluorescence staining

Paraffin-embedded lung tissue sections were deparaffinized with xylene and rehydrated. Antigen retrieval was employed before blocking in PBS with 10% normal goat serum, 0.1% BSA, 0.3% TX-100. The antigen retrieval solution is Tris-EDTA buffer (10 mM Tris Base, 1 mM EDTA solution, 0.05% Tween 20, pH 9.0). The dilutions for rabbit against NAMPT (Bethyl Laboratories, Inc. catalog# A300-372A) and mouse against von Willebrand factor (VWF, catalog# MA5-14029, Invitrogen) are 1:100. Secondary antibodies for NAMPT and VWF were Alexa Fluor® 593 Donkey anti-Rabbit IgG antibody (1:500) and Alexa Fluor® 593 goat anti-mouse IgG antibody (1:500), respectively. An anti-fade mounting media with DAPI (Life Science Inc) was used to fix the coverslip to a slide. The slides were examined using a a ZEISS Z1 AxioObserver fluorescence microscope (Carl Zeiss, Oberkochen, Germany).

### Statistical analysis

Results are shown as mean ± SEM. Experiments repeated at least three times and Graphpad Prism 4.0 software was used for statistical analysis. Normality of the data was assessed with Bartlett’s test. ANOVAs and Kruskal-Wallis tests were used for normal and non-normal data respectively. Tukey, Sidak and Dunn’s post hoc tests were used for pair-wise comparison. Samples were excluded if they failed Grubbs’ outlier test. p < 0.05 was considered statistically significant.

## Supplementary information

Supplemental Figure

## Data Availability

The datasets generated during and/or analyzed during the current study are available from the corresponding author on reasonable request.
