# Supplementary material for: MicroRNA410 Inhibits Pulmonary Vascular Remodeling via Regulation of Nicotinamide Phosphoribosyltransferase
Source: Sci Rep. 2019 Jul 9;9:9949. doi: 10.1038/s41598-019-46352-z (PMC6616369; doi:10.1038/s41598-019-46352-z)
Supplement: Supplementary file 1 — Supplemental Figure [file 41598_2019_46352_MOESM1_ESM.pdf]

MicroRNA410 Inhibits Pulmonary Vascular Remodeling via Regulation of Nicotinamide phosphoribosyltransferase

Hui Gao[1, 2], Jiwang Chen[1], Tianji Chen[3], Yifang Wang[1], Yang Song[4], Yangbasai Dong[1], Shuangping Zhao[1], Roberto F. Machado[1, 5]

[1]Department of medicine, University of Illinois at Chicago, Chicago, IL 60612; [2]Department of Obstetrics and Gynecology, Union Hospital, Tongji Medical College, Huazhong University of Science and Technology, Wuhan 430022, China; [3]Department of Pediatrics, University of Illinois at Chicago, Chicago, IL 60612; [4]Institute for Genome Sciences, University of Maryland School of Medicine, Baltimore, MD, 21201; [5]Division of Pulmonary, Critical Care, Sleep, and Occupational Medicine, Department of Medicine, Indiana University, Indianapolis, IN 46202.

Supplemental Information

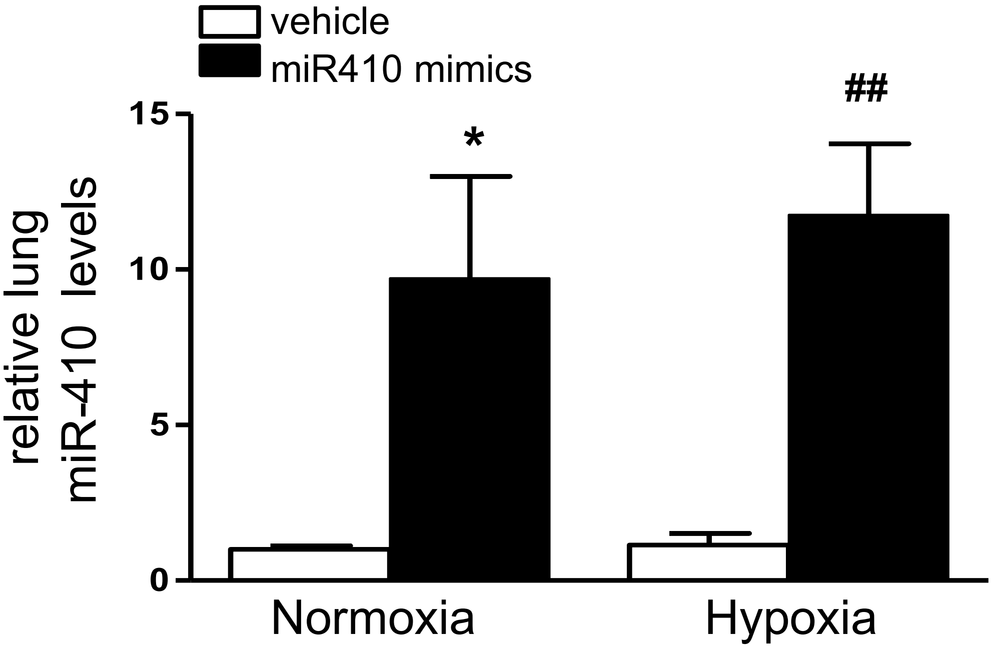

Supplemental Figure 1: Delivery of miR410 mimics increases lung miR410 levels. When compared to control, retro orbital delivery of micro RNA 410 mimic significantly increased the lung expression of miR410 in both normoxia and hypoxia.
